# The epigenetic signature of subcutaneous fat cells is linked to altered expression of genes implicated in lipid metabolism in obese women

**DOI:** 10.1186/s13148-015-0126-9

**Published:** 2015-09-08

**Authors:** Peter Arner, Indranil Sinha, Anders Thorell, Mikael Rydén, Karin Dahlman-Wright, Ingrid Dahlman

**Affiliations:** Lipid laboratory, Department of Medicine, Huddinge, Karolinska Institutet, Stockholm, S-141 86 Sweden; Department of Biosciences and Nutrition, Karolinska Institutet, Stockholm, S-141 83 Sweden; Department of Clinical Sciences, Danderyds Hospital, Karolinska Institutet, Stockholm, Sweden; Department of Surgery, Ersta Hospital, Stockholm, Sweden; SciLifeLab, Science for Life Laboratory, S-171 65 Solna, Sweden

**Keywords:** Adipocytes, Lipolysis, Adipogenesis, Epigenetics

## Abstract

**Background:**

Obesity is associated with changes in fat cell gene expression and metabolism. What drives these changes is not well understood. We aimed to explore fat cell epigenetics, i.e., DNA methylation, as one mediator of gene regulation, in obese women. The global DNA methylome for abdominal subcutaneous fat cells was compared between 15 obese case (BMI 41.4 ± 4.4 kg/m^2^, mean ± SD) and 14 never-obese control women (BMI 25.2 ± 2.5 kg/m^2^). Global array-based transcriptome analysis was analyzed for subcutaneous white adipose tissue (WAT) from 11 obese and 9 never-obese women. Limma was used for statistical analysis.

**Results:**

We identified 5529 differentially methylated DNA sites (DMS) for 2223 differentially expressed genes between obese cases and never-obese controls (false discovery rate <5 %). The 5529 DMS displayed a median difference in beta value of 0.09 (range 0.01 to 0.40) between groups. DMS were under-represented in CpG islands and in promoter regions, and over-represented in open sea-regions and gene bodies. The 2223 differentially expressed genes with DMS were over-represented in key fat cell pathways: 31 of 130 (25 %) genes linked to “adipogenesis” (adjusted *P* = 1.66 × 10^−11^), 31 of 163 (19 %) genes linked to “insulin signaling” (adjusted *P* = 1.91 × 10^−9^), and 18 of 67 (27 %) of genes linked to “lipolysis” (*P =* 6.1 × 10^−5^). In most cases, gene expression and DMS displayed reciprocal changes in obese women. Furthermore, among 99 candidate genes in genetic loci associated with body fat distribution in genome-wide association studies (GWAS); 22 genes displayed differential expression accompanied by DMS in obese versus never-obese women (*P* = 0.0002), supporting the notion that a significant proportion of gene loci linked to fat distribution are epigenetically regulated.

**Conclusions:**

Subcutaneous WAT from obese women is characterized by congruent changes in DNA methylation and expression of genes linked to generation, distribution, and metabolic function of fat cells. These alterations may contribute to obesity-associated metabolic disturbances such as insulin resistance in women.

**Electronic supplementary material:**

The online version of this article (doi:10.1186/s13148-015-0126-9) contains supplementary material, which is available to authorized users.

## Background

Obesity is linked to metabolic complications including insulin resistance (IR) and type 2 diabetes (T2D). Adiposity and development of systemic IR are associated with an interrelated set of adaptions in white adipose tissue (WAT). The ability of catecholamines, the major lipolytic hormones in man, to stimulate lipolysis is blunted in obese subjects [1]. Furthermore, the turnover of adipocyte lipids is decreased in obesity [2]. These metabolic alterations may retain lipids in fat cells and thereby contribute to WAT mass expansion. In addition, the morphology of WAT can influence fat cell lipolysis [3]. Thus as reviewed [4], a phenotype characterized by few but large adipocytes (hypertrophy) is linked to IR.

The cellular adaption of WAT to adiposity is accompanied by major changes in gene expression reflecting both metabolic adaptions of the fat cells, and changes in the tissue as a whole, e.g., fibrosis and inflammation [5, 6]. Through large genome-wide association studies (GWAs), a number of susceptibility genes for obesity and related metabolic disturbances have been mapped, but together they explain no more than a minor proportion of the heritability/variation in these phenotypes, and the culprit genes are in most cases unknown [7–10].

Epigenetic modifications, such as DNA methylation and histone modifications, constitute an additional layer regulating gene expression and thus effecting phenotypes and the development of various states of disease [11]. DNA methylation mainly occurs in the context of CG dinucleotides (CpGs) and has traditionally been associated with gene repression [12]. Global DNA methylomes of human subcutaneous WAT have been related to BMI, body fat distribution, weight loss, and T2D [13–17]. In general, the reported absolute differences in DNA methylation at specific CpG sites between groups have been small, from a few percent up to 10 % [14, 16], and with few exceptions not systematically related to gene expression (e.g., [14, 17, 18]).

The interpretation of differences in DNA methylation profiles for tissues is complicated by the fact that the epigenetic profile can differ substantially between various cell types within a tissue [19]. WAT contains many different cell types of which adipocytes comprise only 20–40 % [16]. Thus, the DNA methylome of non-fat cells can mask differentially DNA methylated sites (DMS) in fat cells. In addition, obesity is associated with altered cellular composition of WAT, e.g., infiltration with inflammatory cells [20]. Changes in the WAT DNA methylome may therefore reflect altered cellular composition rather than true DMS in a specific cell type. In a recent study, we performed DNA methylation profiling on isolated fat cells in order to avoid the confounding effect of mixed cell populations [21]. We compared post-obese women investigated 2 years after bariatric surgery with never-obese controls and reported a number of epigenetic changes in fat cell DNA methylation. In the present study, we report fat cell epigenetic signatures and WAT global transcriptome profiles in obese cases and never-obese control women in order to define DMS that could regulate fat cell gene expression and metabolic adaption to obesity. We report that several genes in pathways involved in adipogenesis, insulin signaling, and lipolysis display DMS accompanied by differential gene expression in obese women. Furthermore, candidate genes for fat distribution identified through GWAs are enriched for DMS.

## Results

### Clinical characteristics of subjects

Clinical characteristics of the included cohorts are detailed in Table 1. Compared with the never-obese controls, the obese individuals displayed significantly higher BMI, plasma insulin, HOMA-IR, triglycerides and diastolic blood pressure. Although systolic blood pressure displayed a trend to be higher in the obese group, the difference was not significant. Finally, the mean fat cell volume was significantly larger in the obese cases than the never-obese controls. There was no significant difference in age between the groups.

**Table 1:** Clinical characteristics of subjects

	Never-obese	Obese^a^	*P* ^b^
*n*	14	15	
Age (years)	45 ± 11	46 ± 11	0.93
Weight (kg)	69 ± 7	115 ± 11	3.4 × 10^−13^
BMI (kg/m^2^)	25.2 ± 2.5	41.4 ± 4.5	4.1 × 10^−12^
Waist to hip ratio	0.85 ± 0.06	0.98 ± 0.06	5.0 × 10^−6^
Systolic blood pressure (mmHg)	123 ± 19	138 ± 22	0.073
Diastolic blood pressure (mmHg)	74 ± 6	85 ± 9	6.0 × 10^−4^
P-Glucose (mmol/l)	5.1 ± 0.4	5.7 ± 1.2	0.053
P-Insulin (mU/l)	4.6 ± 2.3	16.0 ± 10.3	3.7 × 10^−4^
P-Cholesterol (mmol/l)	4.7 ± 1.0	4.9 ± 0.7	0.57
P-HDL Cholesterol (mmol/l)	1.5 ± 0.4	1.1 ± 0.3	0.0058
P-Triglycerides (mmol/l)	0.86 ± 0.72	1.67 ± 0.92	0.013
P-NEFA (mmol/l)	0.57 ± 0.17	0.83 ± 0.16	2.4 × 10^−4^
P-Apolipoprotein B (g/l)	0.83 ± 0.25	0.96 ± 0.25	0.19
P-Apolipoprotein A1 (g/l)	1.39 ± 0.22	1.19 ± 0.24	0.028
Mean fat cell volume (pl)	443 ± 169	994 ± 184	5.5 × 10^−9^

^a^Three of the obese women had type 2 diabetes, of which two were treated with diet plus metformin, and one woman with diet alone. Nine were treated for hypertension and one patient had stable multiple sclerosis and did not receive any drugs

^b^Comparison of control and obese group with unpaired *t* test. Values are mean ± SD

In the subset of samples used for global transcriptome analysis, the mean BMI among the 11 obese cases was 42 ± 5 kg/m^2^ and in the never-obese controls 25 ± 2 kg/m^2^. The mean age in both groups was 49 years. The subjects included for global transcriptome analysis did not differ significantly in phenotype from their corresponding overall group. In the validation cohort, the mean BMI among the 24 obese cases was 40 ± 7 kg/m^2^ and in the 25 never-obese controls, 24 ± 2 kg/m^2^. The mean age in both groups was 43 years.

### Global pattern of adipocyte CpG methylation in never-obese and obese women

**Fig. 1:**
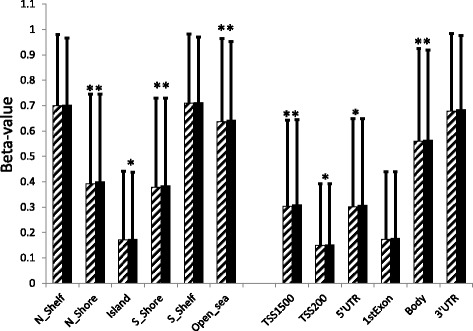
DNA methylation landscape in obese cases versus never-obese control women. After filtering 319,596 CpG probes were mapped to genome regions based on Illumina annotation. We calculated the average level of DNA methylation within the obese (*black bars*) and never-obese (*hatched bars*) groups stratified on genome region in relation to CpG content (*left*), and functional gene regions (*right*). TSS1500; within 1500 basepairs of transcriptional start site (TSS). TSS200; within 200 basepairs of TSS. *P < 0.05; **P < 0.01

The average degree of DNA methylation, i.e., the average beta value for the 319,596 analyzed probes, was higher in fat cells from obese cases (0.425 ± 0.366, mean ± SD) as compared to never-obese controls (0.420 ± 0.375) (*P =* 1.3 × 10^−7^). The average level of DNA methylation stratified by genome region in relation to CpG content and functional parts of genes is shown in Fig. 1. The average DNA methylation of CpG sites located in open seas, CpG islands, and surrounding shore regions was significantly higher in obese as compared to never-obese women whereas no significant difference was observed in shelf regions. The average DNA methylation of CpG sites located in 5′ regions of genes and in gene bodies was significantly higher in obese cases as compared to never-obese controls, whereas there was no significant difference in the 1st exons and 3′UTR regions. The mean within-region absolute difference in DNA methylation between groups was small, in all cases less than 1 %.

**Fig. 2:**
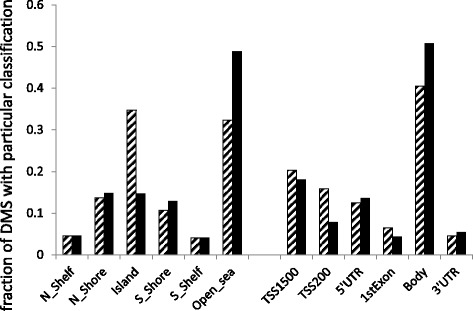
Genomic distribution of DMS between obese and never-obese women in relation to CpG content (*left*) and functional parts of genes (*right*). The genomic distribution of 5529 DMS between obese and never-obese women (*black bars*) (FDR 1 %) were compared to all 319,596 analyzed CpG probes (*hatched bars*). TSS1500; within 1500 basepairs of transcriptional start site (TSS). TSS200; within 200 basepairs of TSS

Among 319,596 analyzed CpG sites, there were 32,724 DMS in fat cells between obese cases versus never-obese controls according to Limma and applying a FDR <1 % (Additional file 1: Table S1). We focused the subsequent analysis on the 23,576 DMS linked to genes. Global transcriptome analysis identified 3878 differentially expressed genes in WAT between obese cases and never-obese controls applying thresholds FDR 5 % and fold change 20 % (Additional file 1: Table S2); 2546 of these genes were expressed at higher levels in obese women. We did not have enough material to perform transcriptome analysis on isolated fat cells. However, since the expression of genes involved in metabolism often display enriched expression in fat cells as compared to stroma cells, we believe that the WAT transcriptome data provide valid information about differential gene expression of relevance also for fat cells. Next, we compared the 23,576 DMS with the 3878 differentially expressed genes and identified 5529 DMS associated with 2223 differentially expressed genes between obese cases and never-obese controls (Additional file 1: Table S3). The 5529 DMS displayed a median difference in beta value of 0.09 (range 0.01 to 0.40) between the obese cases and never-obese controls. The genomic distribution of the 5529 DMS, as compared to all 319,596 analyzed probes, is shown in Fig. 2 in relation to CpG content and functional parts of genes. DMS were under-represented in CpG islands and over-represented in open sea-regions. DMS were under-represented in promoter regions (TSS1500, TSS200) and over-represented in gene bodies. We related CpG methylation at individual DMS to gene expression (Table 2). DMS with inverse association to gene expression were modestly (58 %) over-represented in the 5′ regions of genes (i.e., TSS1500, TSS200, 5′UTR, and first exon) as compared to DMS with directionally consistent change in DNA methylation and expression comparing obese versus never-obese women. DMS in gene bodies and 3′UTR regions displayed an equal distribution of negative and positive associations between DNA methylation and gene expression.

**Table 2:** Relationship between CpG methylation and gene expression for DMS

	Inverse^a^	Same direction^b^
TSS1500	543	455
TSS200	256	177
5′UTR	440	310
1stExon	153	86
Body	1365	1440
3′UTR	145	158

^a^Inverse association between DNA methylation and gene expression in obese versus never-obese women

^b^DNA methylation and gene expression display same directionally consistent change in obese versus never-obese women

### DMS in insulin signaling, adipogenesis, and lipolysis pathways

The 2223 differentially expressed genes associated with DMS were analyzed for over-representation of specific WikiPathways as compared to all genes in the human genome using Webgestalt. The most significantly over-represented pathways include focal adhesion, immune response, adipogenesis, and insulin signaling (Table 3). Of particular interest, 31 of 130 (25 %) of genes linked to “adipogenesis” displayed DMS and differential gene expression (adjusted *P* = 1.66 × 10^−11^); the corresponding proportion for “insulin signaling” was 31 of 163 (19 %) (adjusted *P* = 1.91 × 10^−9^), and for “fatty acid biosynthesis” 12 of 29 (41 %) (adjusted *P* = 3.99 × 10^−8^). Gene expression levels and DNA methylation status for a selection of genes in these pathways are shown in Table 4. A detailed list can be found in Additional file 1: Table S4. In most cases, differential gene expression and DMS display reciprocal changes in obese compares to control women, e.g., expression of *PPARG* and *PPARGC1A* was lower in obese women compared to controls whereas multiple CpG sites in these genes, in particular in the promoter regions (TSS1500), displayed higher methylation in the obese women compared to controls.

**Table 3:** Pathways enriched for differentially expressed genes with DMS

	Number of genes			
Wikipathway	Observed	Expected	Total	Adjusted *P*
Focal adhesion	47	9.25	185	1.52 × 10^−18^
Adipogenesis	31	6.5	130	1.66 × 10^−11^
Regulation of toll-like receptor signaling	33	7.7	154	4.77 × 10^−11^
Integrated Pancreatic Cancer Pathway	35	9.05	181	1.73 × 10^−10^
MAPK signaling pathway	33	8.25	165	2.12 × 10^−10^
B Cell Receptor Signaling Pathway	26	5.7	114	1.21 × 10^−9^
TCR Signaling Pathway	25	5.3	106	1.21 × 10^−9^
Insulin Signaling	31	8.15	163	1.91 × 10^−09^
Muscle cell TarBase	55	21.21	424	1.91 × 10^−09^
Regulation of Actin Cytoskeleton	30	7.85	157	3.00 × 10^−09^
DNA damage response	22	4.38	89	2.97 × 10^−09^
Integrin-mediated cell adhesion	23	4.97	101	6.25 × 10^−09^
AGE-RAGE pathway	19	3.74	76	3.36 × 10^−08^
IL-3 Signaling Pathway	16	2.66	54	3.68 × 10^−08^
Fatty Acid Biosynthesis	12	1.43	29	3.99 × 10^−08^

**Table 4:** Selected differentially expressed genes with DMS in adipogenesis, insulin signaling, and fatty acid biosynthesis pathways

	Expression						DNA methylation							
	Obese		Control		Obese vs control	Adjusted *P* ^a^	Probes	Gene_region	Obese		Control		Obese vs control	Adjusted *P* ^a^
	Average	SD	Average	SD					Average	SD	Average	SD		
Adipogenesis														
KLF15	143	41	253	54	0.57	4.78E−04	cg00540067	5′UTR	0.53	0.09	0.35	0.06	0.18	6.01E−05
							cg27639142	5′UTR	0.33	0.08	0.18	0.06	0.15	1.94E−04
							cg01031983	Body	0.25	0.07	0.13	0.04	0.12	4.85E−05
							cg21468971	Body	0.84	0.06	0.92	0.02	−0.08	2.32E−04
							cg14339848	3′UTR	0.47	0.05	0.37	0.05	0.10	9.90E−04
KLF5	33	4	45	9	0.73	5.16E−03	cg09338033	1stExon	0.06	0.02	0.04	0.01	0.02	1.89E−04
							cg14281591	Body	0.87	0.06	0.92	0.02	−0.05	6.75E−03
PLIN2	506	102	408	53	1.24	3.62E−02	cg03885527	Body	0.84	0.04	0.9	0.03	−0.06	4.18E−03
PPARA	105	13	147	25	0.72	5.54E−04	cg10543624	Body	0.74	0.09	0.86	0.05	−0.12	2.31E−04
PPARG	1607	142	2096	258	0.77	4.08E−04	cg01412654	TSS1500	0.47	0.08	0.33	0.06	0.14	1.87E−04
							cg18063278	TSS1500	0.28	0.09	0.15	0.05	0.13	7.36E−04
							cg25929976	TSS1500	0.24	0.07	0.13	0.04	0.11	1.21E−04
							cg16827534	5′UTR	0.31	0.13	0.15	0.05	0.16	7.11E−04
							cg16197186	5′UTR	0.91	0.03	0.84	0.05	0.07	2.62E−04
							cg10499651	Body	0.23	0.11	0.09	0.02	0.14	8.70E−05
PPARGC1A	58	9	96	31	0.61	2.36E−03	cg11270806	TSS1500	0.18	0.09	0.08	0.02	0.10	3.66E−04
							cg27461259	TSS1500	0.35	0.07	0.16	0.04	0.19	5.36E−06
							cg27514608	TSS1500	0.21	0.11	0.08	0.02	0.13	6.43E−05
Insulin signaling														
AKT2	1021	104	1302	205	0.78	3.57E−03	cg14309246	TSS1500	0.23	0.09	0.13	0.03	0.10	5.03E−03
							cg25333225	TSS1500	0.17	0.05	0.11	0.02	0.06	9.61E−04
							cg13351352	Body	0.35	0.12	0.18	0.07	0.17	6.22E−04
							cg15153957	3′UTR	0.14	0.13	0.06	0.02	0.08	4.54E−03
INSR	192	19	244	42	0.79	6.85E−03	cg00428638	Body	0.29	0.09	0.16	0.08	0.13	3.34E−03
							cg09779027	Body	0.33	0.09	0.16	0.05	0.17	3.99E−05
							cg10148591	Body	0.25	0.08	0.13	0.03	0.12	2.11E−04
							cg23845936	Body	0.63	0.08	0.43	0.08	0.20	4.44E−05
IRS1	121	24	181	63	0.67	3.46E−02	cg00727310	1stExon	0.35	0.09	0.2	0.04	0.15	1.03E−04
							cg04129548	1stExon	0.39	0.09	0.22	0.07	0.17	1.88E−04
							cg13008631	1stExon	0.36	0.07	0.17	0.06	0.19	1.11E−05
							cg04751089	3′UTR	0.14	0.07	0.04	0.02	0.10	1.20E−05
							cg00305996	3′UTR	0.55	0.13	0.3	0.11	0.25	1.78E−04
IRS2	115	19	184	28	0.62	2.99E−05	cg25312054	1stExon	0.67	0.09	0.49	0.08	0.18	1.24E−04
							cg03337886	1stExon	0.37	0.07	0.21	0.06	0.16	2.99E−05
							cg10488031	1stExon	0.35	0.03	0.25	0.05	0.10	1.17E−03
							cg05514401	1stExon	0.69	0.09	0.57	0.09	0.12	1.00E−02
							cg01569664	Body	0.49	0.12	0.31	0.08	0.18	6.38E−03
							cg12085119	Body	0.53	0.08	0.34	0.09	0.19	9.22E−05
							cg13539803	Body	0.92	0.03	0.95	0.01	−0.03	2.83E−03
							cg20445402	Body	0.76	0.14	0.44	0.11	0.32	2.45E−05
							cg24526103	Body	0.29	0.14	0.11	0.04	0.18	7.59E−04
							cg25924746	Body	0.39	0.13	0.16	0.06	0.23	3.97E−05
SGK2	265	46	208	57	1.28	3.45E−02	cg04420889	TSS1500	0.44	0.16	0.2	0.1	0.24	1.11E−03
							cg21685427	TSS1500	0.36	0.08	0.26	0.06	0.10	3.13E−03
							cg06600331	TSS200	0.39	0.07	0.29	0.06	0.10	4.91E−03
							cg06796271	TSS200	0.26	0.11	0.11	0.03	0.15	1.18E−04
SLC2A4	120	27	304	85	0.39	5.34E−06	cg03670302	3′UTR	0.78	0.06	0.65	0.1	0.13	1.38E−03
Fatty acid biosynthesis														
ACACA	206	38	301	83	0.69	4.23E−03	cg01760189	TSS1500	0.51	0.11	0.31	0.08	0.20	1.83E−04
							cg20778688	TSS1500	0.69	0.09	0.53	0.07	0.16	3.11E−04
							cg07375836	5′UTR	0.19	0.04	0.13	0.06	0.06	8.52E−03
							cg16822666	5′UTR	0.19	0.08	0.08	0.04	0.11	3.98E−04
							cg06026545	Body	0.14	0.07	0.08	0.02	0.06	3.65E−03
							cg07834934	Body	0.65	0.05	0.72	0.05	−0.07	5.96E−03
							cg08013737	Body	0.43	0.1	0.29	0.07	0.14	3.07E−03
							cg15939920	Body	0.87	0.05	0.92	0.02	−0.05	1.48E−03
							cg26100256	Body	0.83	0.05	0.72	0.03	0.11	4.43E−05
ACACB	1765	158	2720	440	0.65	1.88E−05	cg12178147	TSS1500	0.47	0.13	0.21	0.08	0.26	2.81E−05
							cg23921871	1stExon	0.59	0.09	0.46	0.08	0.13	5.87E−03
							cg06002638	Body	0.49	0.08	0.36	0.05	0.13	8.85E−04
ACLY	428	66	773	494	0.55	3.41E−02	cg19443920	TSS1500	0.12	0.06	0.06	0.01	0.06	1.50E−03
							cg12641024	5′UTR	0.37	0.16	0.17	0.05	0.20	1.09E−03
							cg14583225	5′UTR	0.34	0.09	0.18	0.06	0.16	8.03E−05
							cg27470486	5′UTR	0.13	0.08	0.06	0.03	0.07	2.61E−03
							cg01761362	Body	0.37	0.16	0.16	0.07	0.21	6.64E−04
PECR	392	64	597	120	0.66	5.14E−04	cg10881745	Body	0.71	0.09	0.44	0.09	0.27	1.02E−05

^a^Adjusted *P* comparing groups in Limma (in DNA methylation analysis adjusting for age)

Fat cell lipolysis is a key pathway to examine for epigenetic impact since dysregulated fat cell lipolysis has been inked to both increased fat storage, via blunted catecholamine-induced lipolysis, and IR by enhanced spontaneous (basal) lipolysis. The lipolysis pathway is not listed as a pathway in public databases and was therefore not included in the analysis above. We defined in the following analysis, genes included in the lipolytic pathway as the genes listed in the comprehensive review by Lafontan and Langin [22]. Among 67 lipolysis genes, 18 displayed differential expression and DMS between obese and never-obese women (*P =* 6.1 × 10^−5^). Lipolysis genes with DMS are shown in Table 5 and include *ABHD5* (the coactivator of *ATGL*), *ADCY2*, *ADRB1*, *CIDEA*, and *PLIN2*.

**Table 5:** Lipolysis genes displaying differentially expression accompanied by DMS in obese women compared to controls

	Expression						DNA methylation							
	Obese		Control		Obese vs control	Adjusted *P* ^a^	Probes	Region	Obese		Control		Obese vs control	Adjusted *P* ^a^
	Av.	SD	Av.	SD					Av.	SD	Av.	SD		
ABHD5	808	64	1327	343	0.61	9.76E−05	cg24595152	Body	0.57	0.06	0.4	0.1	0.17	2.04E−04
ADCY2	24	6	34	8	0.71	1.08E−02	cg07176385	Body	0.69	0.07	0.6	0.05	0.09	9.27E−03
							cg27629673	Body	0.78	0.07	0.86	0.04	−0.08	8.43E−03
							cg12378867	3′UTR	0.27	0.1	0.16	0.05	0.11	4.90E−03
ADCY6	302	36	381	53	0.79	3.62E−03	cg22689690	TSS1500	0.04	0.02	0.02	0.01	0.02	1.61E−03
							cg00160359	5′UTR	0.78	0.06	0.7	0.04	0.08	2.89E−03
							cg11661914	5′UTR	0.26	0.09	0.14	0.05	0.12	1.33E−03
							cg24092939	5′UTR	0.18	0.1	0.07	0.03	0.11	8.58E−04
							cg25196508	5′UTR	0.28	0.13	0.11	0.07	0.17	1.12E−03
							cg26266429	Body	0.19	0.07	0.1	0.07	0.09	8.53E−04
ADCY7	108	18	63	19	1.72	1.76E−04	cg23580000	1stExon	0.86	0.07	0.93	0.03	−0.07	1.85E−03
							cg16548911	Body	0.58	0.08	0.68	0.07	−0.1	6.66E−03
ADRB1	57	16	79	23	0.72	2.80E−02	cg13848598	1stExon	0.2	0.07	0.1	0.03	0.1	7.02E−04
							cg14826456	1stExon	0.08	0.03	0.05	0.02	0.03	1.45E−03
CIDEA	164	44	567	187	0.29	1.01E−06	cg14976646	TSS1500	0.08	0.03	0.05	0.01	0.03	1.34E−03
							cg19883905	TSS1500	0.05	0.02	0.03	0.01	0.02	4.68E−03
							cg14923652	3′UTR	0.93	0.03	0.85	0.04	0.08	4.32E−05
CIDEC	1857	174	2597	265	0.71	2.42E−05	cg03604278	TSS1500	0.3	0.09	0.14	0.02	0.16	1.20E−05
							cg07222243	5′UTR	0.28	0.07	0.13	0.05	0.15	3.13E−05
EDNRA	327	78	463	116	0.71	9.64E−03	cg17073859	TSS1500	0.48	0.05	0.37	0.04	0.11	1.27E−04
							cg00379467	TSS200	0.23	0.07	0.12	0.04	0.11	5.01E−04
							cg00974629	TSS200	0.38	0.07	0.25	0.04	0.13	7.57E−05
							cg05618426	TSS200	0.26	0.09	0.13	0.02	0.13	9.82E−05
EDNRB	618	61	488	81	1.27	3.57E−03	cg07974719	TSS1500	0.31	0.06	0.24	0.03	0.07	9.57E−03
							cg12983394	TSS1500	0.05	0.04	0.02	0.01	0.03	5.52E−03
							cg12120741	1stExon	0.29	0.06	0.19	0.04	0.1	3.77E−04
							cg18210860	Body	0.24	0.12	0.1	0.03	0.14	3.35E−04
GNG7	78	14	112	21	0.69	9.31E−04	cg01286319	5′UTR	0.53	0.1	0.67	0.07	−0.14	1.64E−03
							cg02309655	5′UTR	0.3	0.08	0.19	0.04	0.11	6.22E−03
							cg06371583	5′UTR	0.26	0.06	0.19	0.04	0.07	8.78E−03
							cg08461840	5′UTR	0.54	0.11	0.71	0.09	−0.17	7.26E−04
							cg11906607	5′UTR	0.33	0.1	0.21	0.07	0.12	3.69E−03
							cg13078421	5′UTR	0.09	0.05	0.04	0.01	0.05	3.72E−04
							cg18229071	5′UTR	0.54	0.07	0.64	0.05	−0.1	1.81E−03
							cg18754118	5′UTR	0.34	0.07	0.25	0.03	0.09	2.71E−03
							cg19382697	5′UTR	0.77	0.05	0.84	0.03	−0.07	1.03E−03
							cg19853565	5′UTR	0.15	0.13	0.05	0.02	0.1	1.74E−03
							cg20091384	5′UTR	0.24	0.07	0.16	0.03	0.08	5.42E−03
							cg22023664	5′UTR	0.76	0.09	0.88	0.02	−0.12	2.35E−04
							cg24874003	5′UTR	0.2	0.12	0.07	0.03	0.13	7.32E−04
							cg27176392	5′UTR	0.92	0.02	0.87	0.03	0.05	1.28E−03
IL6R	141	16	103	13	1.36	1.47E−04	cg24346686	TSS1500	0.13	0.04	0.09	0.03	0.04	7.89E−03
							cg04437762	Body	0.67	0.07	0.82	0.05	−0.15	1.94E−05
							cg17001401	Body	0.31	0.08	0.15	0.05	0.16	4.73E−05
							cg25135018	Body	0.76	0.09	0.88	0.05	−0.12	5.63E−04
INSR	192	19	244	42	0.79	6.85E−03	cg00428638	Body	0.29	0.09	0.16	0.08	0.13	3.34E−03
							cg09779027	Body	0.33	0.09	0.16	0.05	0.17	3.99E−05
							cg10148591	Body	0.25	0.08	0.13	0.03	0.12	2.11E−04
							cg23845936	Body	0.63	0.08	0.43	0.08	0.2	4.44E−05
NPR1	265	41	374	44	0.71	2.21E−04	cg07106989	Body	0.9	0.02	0.81	0.04	0.09	4.62E−05
PDE3B	701	119	972	169	0.72	2.00E−03	cg03439703	TSS1500	0.17	0.04	0.11	0.02	0.06	3.83E−04
							cg18222865	TSS1500	0.34	0.06	0.2	0.05	0.14	1.10E−04
							cg21901307	TSS1500	0.1	0.03	0.06	0.01	0.04	1.12E−03
							cg12177909	Body	0.31	0.1	0.19	0.05	0.12	2.74E−03
PDE5A	118	14	90	17	1.31	3.31E−03	cg15191465	TSS1500	0.05	0.01	0.03	0.01	0.02	2.68E−03
							cg19191984	TSS1500	0.08	0.02	0.05	0.01	0.03	3.78E−03
							cg06531595	Body	0.78	0.05	0.71	0.03	0.07	2.47E−03
PLIN2	506	102	408	53	1.24	3.62E−02	cg03885527	Body	0.84	0.04	0.9	0.03	−0.06	4.18E−03
PPARG	1607	142	2096	258	0.77	4.08E−04	cg01412654	TSS1500	0.47	0.08	0.33	0.06	0.14	1.87E−04
							cg18063278	TSS1500	0.28	0.09	0.15	0.05	0.13	7.36E−04
							cg25929976	TSS1500	0.24	0.07	0.13	0.04	0.11	1.21E−04
							cg16197186	5′UTR	0.91	0.03	0.84	0.05	0.07	2.62E−04
							cg16827534	5′UTR	0.31	0.13	0.15	0.05	0.16	7.11E−04
							cg10499651	Body	0.23	0.11	0.09	0.02	0.14	8.70E−05
PRKAR2B	1956	324	2467	178	0.79	3.69E−03	cg03661844	Body	0.95	0.03	0.79	0.11	0.16	5.45E−06
							cg10691109	Body	0.1	0.06	0.04	0.01	0.06	9.79E−05
							cg26104690	Body	0.77	0.08	0.66	0.07	0.11	3.64E−03

*Av* average

^a^ Adjusted *P* comparing groups in Limma (in DNA methylation analysis adjusting for age)

The list of 5529 DMS accompanied with differential expression contains 120 cross-reactive probes (Additional file 1: Table S3). We have not applied any filter based on detection *P* values. If we apply a cutoff filter *P* < 0.01 on the list of 5529 DMS, 24 CpG sites do not pass the test. There were no cross-reactive probes in the list of DMS linked to candidate pathways, e.g., adipogenesis, lipolysis, and insulin signaling. One probe (cg10543624 in *PPARA*) had detection *P* > 0.01.

### DMS of candidate genes in genetic loci linked to adiposity in GWAS

Next, we investigated if candidate genes in genetic loci linked to adiposity in GWAS displayed evidence of epigenetic regulation. Among 150 candidate protein coding genes in 97 genetic loci associated with BMI [10], 20 genes displayed differential expression accompanied by DMS in obese women compared to controls which was slightly more than expected (*P =* 0.045) (Additional file 1: Table S5). Furthermore, among 99 protein coding candidate genes in 69 genetic loci associated with body fat distribution [9], 22 genes displayed differential expression accompanied by DMS in obese versus never-obese control women (*P* = 0.0002). Candidate genes for body fat distribution with DMS in this study included *ADAMTS9, ARL15, C5, CMIP, CPEB4, EYA1, FAM13A, FGF2, GMDS, HLA-DRA, KCNJ2, KLHL31, LY86, MAP3K1, MSC, NLRP3, PEMT, PLCG2, PPARG, TBX15, TFPI,* and *VEGFA* (Additional file 1: Table S6).

### Comparison with DMS in other cohorts

Finally, we examined if the 32,724 obesity-associated DMS in fat cells detected in the present study were identified in WAT in separate cohorts. Among all 32,724 DMS, 1474 sites had been assayed in WAT from an independent set of obese and non-obese women using the Illumina 27K array. Ninety of 1474 CpG sites were differentially methylated between obese and non-obese women (*P* < 0.05, one-sided test); for 66 of 90 DMS the association was directionally consistent with the results on the 450K array (Additional file 1: Table S7; Additional file 2: Figure S1). The mean absolute level of methylation at specific CpG sites, measured as beta value, differed substantially between fat cells (450K array) and WAT (27K array); this could be due to differences in methylation between fat cells and WAT stroma cells. This is supported by the fact that the absolute difference in DNA methylation at specific CpG sites between obese versus non-obese women tend to be larger in fat cells as compared to WAT.

We also mapped the 32,724 DMS from the present study to a number of reported DNA methylation profiling studies on WAT applying the 450K platform. Benton MC et al. identified 3601 DMS before versus after weight loss induced by bariatric surgery [16]. Importantly, 1239 DMS described by Benton et al. overlapped with the present study of which 1236 CpG sites displayed directionally consistent difference in methylation in the comparisons before versus after weight loss, and obese versus never-obese women (Additional file 1: Table S8; Additional file 2: Figure S2). Rönn et al. identified 39,533 CpG sites whose methylation in WAT of women associated with BMI. BMI-associated CpG sites (8079) overlap with the present study of which 7876 displayed directionally consistent difference in methylation (Additional file 1: Table S9) [17]. Furthermore, Nilsson et al. identified 15,627 DMS in WAT associated with T2D [14]. DMS (2885) overlapped with the present study, of which 2630 DMS displayed directionally consistent difference in methylation in T2D and obesity (Additional file 1: Table S10) [14]. Overall, the performed comparisons with three other independent analyses support the accuracy of our assay.

## Discussion

Herein we describe the global methylome of isolated human fat cells in relation to adiposity. We find that obesity is associated with a large number of DMS. In particular, several genes in the adipogenesis, insulin signaling, and lipolysis pathways display DMS accompanied by differential gene expression comparing obese and control women. Furthermore, candidate genes for fat distribution from GWAS are enriched for DMS in this study.

The global pattern of DMS in the genome reported here is consistent with, and complements, findings that previously have been reported for human WAT, which contains a number of cell types besides fat cells. We observed a slightly higher global mean DNA methylation in obese women, while Benton et al. have reported significantly higher DNA methylation before compared to after weight loss for all gene regions in subcutaneous WAT [16]. This implies that higher DNA methylation in obese is secondary to obesity and reversed upon weight loss. In agreement with this, we observe higher DNA methylation in obese women when comparing the global fat cell DNA methylome pattern between obese (present study) and post-obese women [21]. The relative fat cell turnover rate is not affected by obesity [23] and it is therefore unlikely that differences in fat cell age explain DNA hypermethylation in obese women. The absolute difference in global DNA methylation between groups was small, but in the same order of magnitude as observed in other studies [16, 21]. Future studies are needed to determine the functional significance of observed changes in global DNA methylation.

The genomic distribution of DMS, i.e., relatively few near CpG islands and in the promoter regions, and over-representation in open sea-regions is in agreement with what has been reported in WAT in relation to physical exercise and T2D [18, 24]. CpG sites displaying inverse correlations between DNA methylation and expression of associated genes were over-represented in promoter and 5′ regions of genes, whereas CpG sites showing positive correlations between DNA methylation and expression of associated genes were over-represented in gene bodies and 3′UTRs, which is in agreement with the literature on WAT epigenetics [24]. In fact, whereas DNA methylation traditionally has been considered to be a repressor of gene expression, methylation of CpG sites in gene bodies often show a positive correlation with active transcription [25]. Previous WAT DNA methylome studies report smaller absolute difference in methylation at specific CpG sites between clinical groups compared to what we observe in fat cells. The median delta-beta was 0.09 in the present study, whereas the delta-beta rarely was larger than 5 % in WAT between T2D and healthy controls [24]. Twenty-two CpG sites had delta-beta >20 % after versus before weight loss, whereas 773 CpG sites reached this threshold in the present study [16]. It is possible that differences are due to that we investigate isolated fat cells, whereas previous studies were performed on WAT pieces, which contain a number of additional cell types besides the fat cells. The latter may also explain the limited overlap with reported DMS associated with adiposity or responding to weight loss [13, 15, 16].

Are the observed variations in DMS of biological significance? Although we did not perform direct molecular studies, our findings when comparing DMS with gene expression suggest a pathophysiological role. We report over-representation of DMS accompanied by differential expression in genes in key fat cell pathways such as adipogenesis, insulin signaling, and lipolysis. In most cases, adipogenesis, insulin signaling, and lipolysis genes were lower expressed in obese as compared to never-obese control women. Blunted lipolytic response has been linked to obesity [1]. WAT hypertrophy is associated with IR and has been linked to dysregulation of adipogenesis [26]. These pathways are known to be dysregulated in WAT of obese individuals, but the upstream regulation is poorly understood. Our results support the notion of epigenetic dysregulation of adipogenesis, insulin signaling, and lipolysis pathways being present in obese women. Numerous studies have highlighted the importance of epigenetic regulation of adipogenesis in vitro (reviewed in [27]). As far as we know, we here provide the first evidence for epigenetic regulation of fat cell lipolysis. Lipolysis is believed to promote fat storage, whereas epigenetic regulation of all three pathways could contribute to obesity-associated insulin resistance. There is some overlap in pathways with DMS reported in the present study and those previously reported to display differential DNA methylation in response to weight loss, e.g., focal adhesion, and adipogenesis, suggesting that epigenetic dysregulation of these pathways could be reversed upon weight loss [16].

A recent study showed the value of epigenetics as a complement to GWAS to pinpoint candidate genes harboring susceptibility alleles for T2D [28]. Recently, numerous new genetic loci linked to fat distribution have been mapped [9]. Candidate genes in these loci have been shown to display enriched expression in WAT as compared to other organs and have implied that adipogenesis and insulin signaling pathways are involved in the regulation of fat distribution. Our results complement these findings. We show a strong over-representation of fat cell DMS accompanied by differential gene expression of candidate genes for fat distribution. Among these genes, *PPARG, TBX15*, and *PEMT* have previously been implicated in adipogenesis and *VEGFA* as well as *FGF2* in angiogenesis [9].

One limitation of the present study is that gene expression was performed on WAT specimen, since we did not have sufficient amounts of isolated fat cells for global transcriptome analysis. Genes involved in fat cell metabolic regulation are usually overexpressed in fat cells as compared to the stroma [5]. Furthermore, we did compare differentially expressed genes in adipocyte pathways in the present study with results from Lee YH et al. who performed global transciptome profiling on adipocytes from obese and non-obese Pima Indians [29]. Their array had limited coverage and they used a very stringent threshold to define differentially expressed genes. Among 85 genes in adipocytes pathways in the present study, 17 were also differentially expressed in the study by Lee YH et al. *(EDNRB, GNG7, NPR1, PDE3B, PRKAR2B, AGPAT2, IRS2, LMNA, NR3C1, NRIP1, EIF4EBP1, ACACB, ECHS1, FASN, HADH, PC, PECR).* Genes overlapping between the studies all showed a directionally consistent change. This confirms that genes in adipocyte pathways are differentially expressed in fat cells of obese compared to lean subjects.

## Conclusions

In conclusion, DMS accompanied by differential expression in genes linked to fat distribution and fat cell metabolism may contribute to abdominal fat storage and obesity-associated IR in women.

## Methods

### Subjects and clinical evaluation

Clinical data are presented in Table 1. Fifteen obese women (BMI >30 kg/m^2^) and 14 never-obese healthy control women (BMI <30 kg/m^2^) were recruited in association with planned visits to our surgical units for gastric by-pass surgery because of obesity and through local advertisement for the purpose of studying WAT factors regulating body weight. Data not shown herein on never-obese women have been reported elsewhere [21]. Four never-obese and 4 obese women were menopausal. All 14 never-obese women were healthy. Three of the obese women had T2D, out of which two were treated with diet and metformin, and one subject with diet alone. Nine of the obese individuals were treated for hypertension. One patient had stable mild multiple sclerosis but did not receive any treatment for this indication. The women undergoing gastric by-pass surgery participated in a trial on the effect of bariatric surgery (NCT01785134 at www.clinicaltrials.gov).

Transcriptome analysis on WAT specimens was conducted for 20 of the above individuals (11 obese and 9 never-obese). For remaining subjects included in this study, we did not have sufficient amount of WAT for transcriptome analysis.

For validation of DMS in an independent cohort, we studied 24 obese otherwise healthy and 25 non-obese healthy women, who have been described previously [30]. The validation was performed on DNA from WAT specimens since we did not have isolated fat cells from this cohort.

### Ethics and consent

The study was approved by the Regional Ethics Committee in Stockholm (2003/326) and all subjects gave their written informed consent to participation.

### Clinical evaluation and WAT sampling

Participants were investigated at 8 AM after an overnight fast. Anthropometric measurements (height, weight, waist and hip circumference, blood pressure) were performed and followed by a venous blood sample. Blood glucose and lipids were analyzed at the hospital’s routine chemistry laboratory. Plasma insulin was measured by ELISA (Mercodia, Uppsala, Sweden) as previously described [31]. Biopsies from the subcutaneous abdominal WAT were obtained by needle aspiration under local anesthesia. WAT samples were thoroughly rinsed in sodium chloride (9 mg/ml).

### Handling of WAT samples and isolation of fat cells

From WAT samples, we isolated the fat cell fraction according to the collagenase procedure [32]. Mean fat cell volume was determined as previously described [33]. Briefly, in adipocyte suspensions, we measured cell sizes by direct microscopy and the mean adipocyte diameter was calculated from measurements of 100 cells. Since adipocytes are spherical in shape, cell volume can be estimated from the diameter. From adipose specimens, 200 μl of packed isolated fat cells and/or 300 mg unfractionated WAT pieces were frozen in liquid nitrogen and kept at −70 °C for subsequent DNA (cells) or RNA (tissue) preparation.

### DNA preparation

Genomic DNA was prepared from fat cells using the QiAamp DNA Mini kit (cat no. 51304, Qiagen, Hilden, Germany). The DNA purity and quality was confirmed by A260/280 ratio >1.8 on a Nanodrop ND-1000 Spectrophotometer (Thermo Fisher Scientific Inc., Waltham, MA, US). The DNA concentration was measured by Qubit (Life technologies, Stockholm, Sweden).

### DNA methylation microarray assays

DNA methylation was analyzed in DNA extracted from fat cells using the Infinium Human Methylation 450 BeadChip assay (Illumina, San Diego, CA, USA). Genomic DNA (500 ng) was bisulfite treated using the EZ DNA methylation kit (Zymo Research, Orange, CA, USA) with the alternative incubation conditions recommended when using the Infinium Methylation Assay. The methylation assay was performed on 4 μl bisulfite-converted genomic DNA at 50 ng/μl according to the Infinium HD Methylation Assay protocol (Part #15019519, Illumina).

For validation of DMS in the independent cohort, bisulphite converted DNA from WAT specimens was hybridized to the Illumina Infinium 27K Human Methylation Beadchip v1.2 using standardized protocols (Illumina). DNA methylation data have been deposited in the National Center for Biotechnology Information Gene Expression Omnibus (GEO; https://www.ncbi.nlm.nih.gov/geo) and are accessible using GEO series accession numbers GSE67024 and GSE24884, respectively. The methylation assays were done at BEA (www.bea.ki.se).

### DNA methylation microarray: bioinformatic analyses

BeadChip images were captured using the Illumina iScan. The raw methylation score for each probe represented as a methylation beta value was calculated using the GenomeStudio Methylation module software (2010.3) [34]. All included samples showed high-quality bisulfite conversion according to Zymo control samples and also passed all GenomeStudio quality control steps based on built in control probes for staining, hybridization, extension, and specificity. For the Infinium Human Methylation 450 BeadChip arrays, we next applied the Bioconductor Lumi package to perform color and quantitative normalization of the DNA methylation data. The BMIQ package was used to adjust the beta values of type 2 design microarray probes into a statistical distribution characteristic of type 1 probes. Beta values were converted to *M* values [*M* = log2(beta/(1 − beta))], a statistically more valid method for conducting differential methylation analysis. As the beta value is easier to interpret biologically, *M* values were reconverted to beta values when describing the results.

The Infinium Human Methylation 450 BeadChip array contains 485,577 probes, which covers 21,231 (99 %) of RefSeq genes. Probes overlapping SNPs can interfere with hybridization. Probes (88,464) containing common SNPs with minor allele frequency (MAF) >10 % according to Illumina were therefore excluded. A further 77,517 CpG probes with SNPs within 10 basepairs from the interrogated CpG sites were excluded. This last filtering step was motivated by our observation that these probes were threefold enriched among probes that displayed the largest variation (standard deviation) in DNA methylation between samples. It is possible that the presence of SNPs inferred with probe hybridization and quantification of DNA methylation. Following the filtering steps, 319,596 probes were taken forward to identify DMS. We used Webgestalt to identify Wikipathways over-represented with DMS as compared to all genes in the human genome [35].

### Transcriptome microarray assay

WAT specimens (100 mg) were disrupted mechanically. From high-quality total RNA, we prepared and hybridized biotinylated complementary RNA to Gene 1.1 ST Arrays, and then washed, stained, and scanned the arrays using standardized protocols (Affymetrix Inc., Santa Clara, CA, USA). The microarray hybridizations were done at BEA (www.bea.ki.se). Subsequent data analyses were performed using the Affymetrix Expression Console version 1.1. The Robust Multi-array analysis algorithm was used for data normalization and calculation of gene expression. To allow comparisons of transcript levels between samples, all samples were subject to an all-probeset scaling-to-target signal of 100.

Among the 33,297 probesets on the Gene 1.1 ST array, we filtered for the 22,371 probesets annotated with a gene symbol. Following exclusion of uncharacterized transcripts labeled LOC or Flj, 21,534 probesets were taken forward for subsequent analysis of differentially expressed genes. When multiple probesets represented the same gene, we show results for the probeset with the highest call; the results (*P* values and fold change in expression) are very similar independently of which probeset is used.

### Statistical analysis

We used the Bioconductor package Limma on methylation *M* values to identify DMS between obese and never-obese women, adjusting for age which is known to influence DNA methylation, and applying the thresholds false discovery rate (FDR) 1 %; we chose a stringent threshold here to avoid false positive [36, 37]. We also used Limma to compare gene expression between the obese and never-obese groups. In transcriptome analysis, we used the thresholds FDR 5 % and fold change 20 % since our experience from previous studies is that by applying these thresholds we can confirm the results by quantitative PCR. Unpaired *t* test was applied to compare average global DNA methylation between the obese and never-obese groups, and in analysis of specific DMS in the validation cohorts.

## Additional files

Additional file 1:
**Additional tables with data on DMS and differentially expressed genes between obese cases versus never-obese controls Table S1.** Differentially methylated DNA sites linked to genes between obese and never-obese women. **Table S2.** Differentially expressed genes between obese and never-obese women. **Table S3.** Differentially DNA methylated sites accompanied by differential expression. **Table S4.** Differentially expressed genes with DMS in Adipogenesis, Insulin Signaling, and Fatty Acid Biosynthesis pathways. **Table S5.** Differentially expressed genes with DMS in candidate genes for BMI according to GWAS. **Table S6.** Differentially expressed genes with DMS in candidate genes for fat distribution according to GWAS. **Table S7.** DMS in WAT in relation to obesity. **Table S8.** DMS in WAT in relation to weight loss (Benton MC et al., Genome Biology 2015). **Table S9.** DMS in WAT in relation to BMI (Rönn T et al., Human Molecular Genetics 2015). **Table S10.** DMS in WAT in relation to T2D (Nilsson E et al., Diabetes 2014). (XLSX 5076 kb) Additional file 2:
**Comparison of obesity associated DMS between fat cells and WAT. Figure S1.** Comparison of obesity associated DMS between fat cells (450 K) and WAT (27 K). **Figure S2.** Comparison of obesity associated DMS in fat cells with weight-loss associated DMS in WAT. (PPTX 71 kb)

## Abbreviations

CpG: CG dinucleotides
FDR: false discovery rate
GWAS: genome-wide association study
IR: insulin resistance
T2D: type 2 diabetes
