# Supplementary material for: The epigenetic signature of subcutaneous fat cells is linked to altered expression of genes implicated in lipid metabolism in obese women
Source: Clin Epigenetics. 2015 Sep 8;7(1):93. doi: 10.1186/s13148-015-0126-9 (PMC4562340; doi:10.1186/s13148-015-0126-9)
Supplement: Additional file 2: — Comparison of obesity associated DMS between fat cells and WAT. Figure S1. Comparison of obesity associated DMS between fat cells (450 K) and WAT (27 K). Figure S2. Comparison of obesity associated DMS in fat cells with weight-loss associated DMS in WAT. (PPTX 71 kb) [file 13148_2015_126_MOESM2_ESM.pptx]

## Slide 1
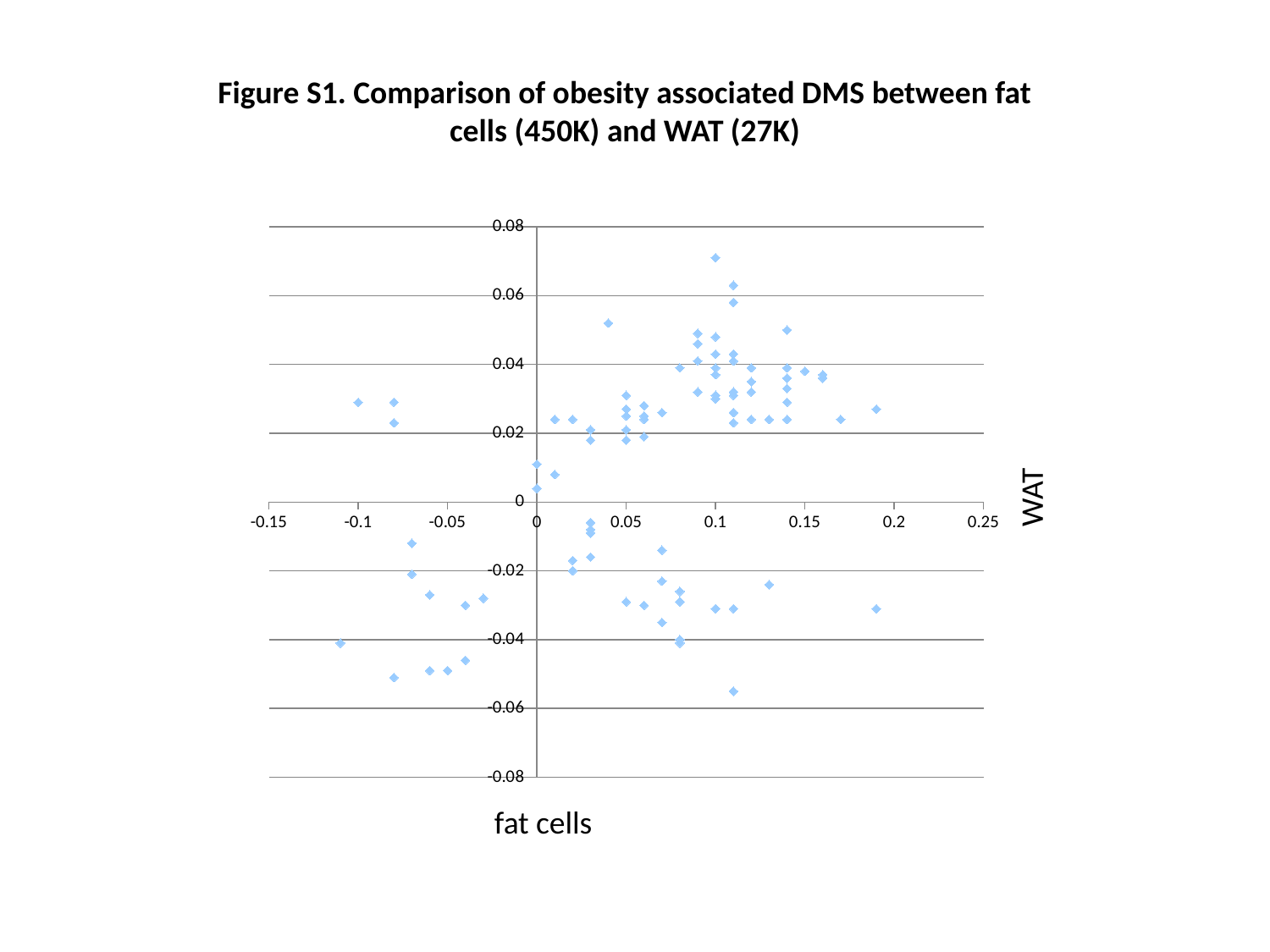

Figure S1. Comparison of obesity associated DMS between fat cells (450K) and WAT (27K)
### Chart
| Category | 27k |
|---|---|WAT
fat cells

## Slide 2
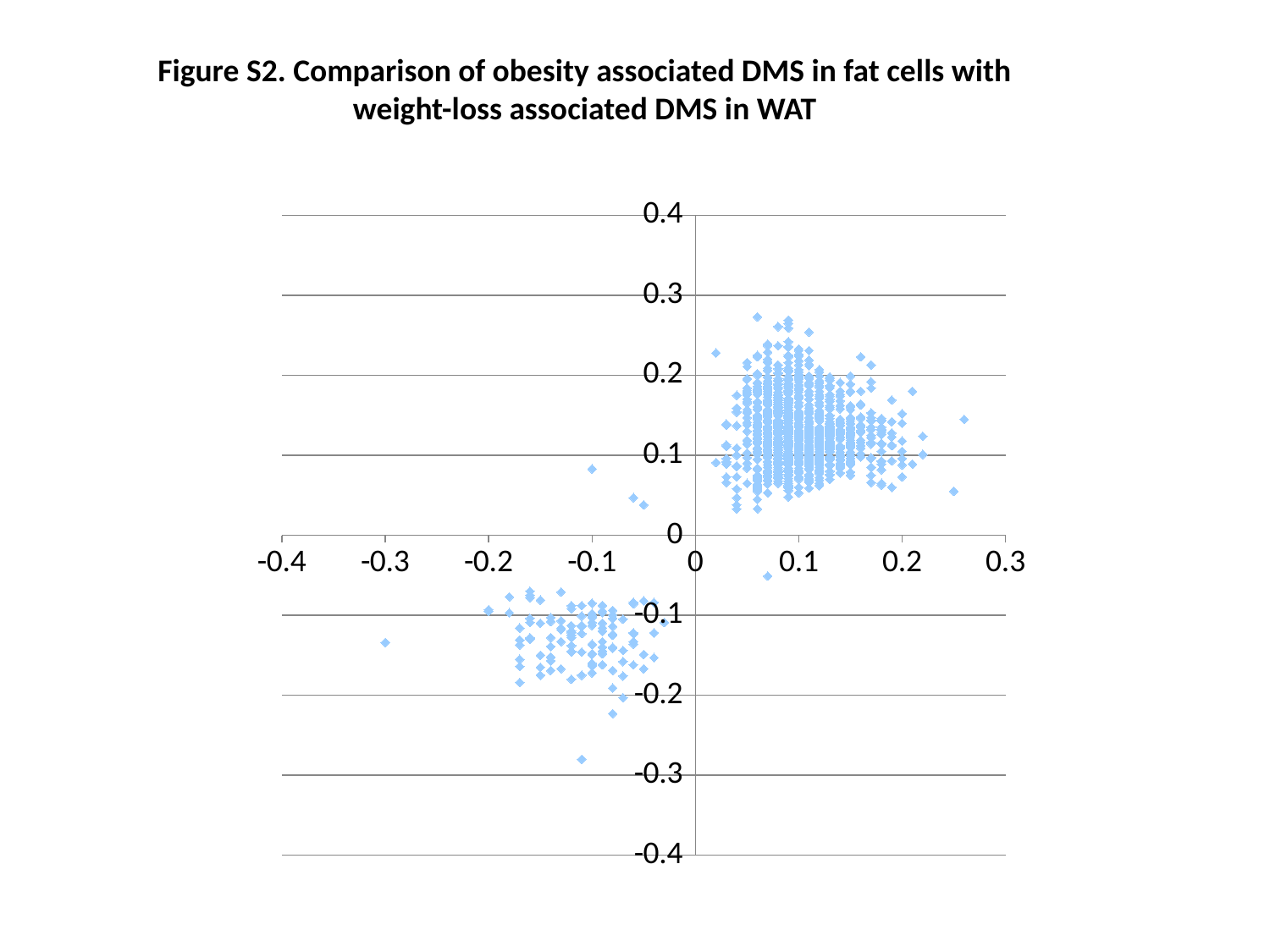

Figure S2. Comparison of obesity associated DMS in fat cells with weight-loss associated DMS in WAT
### Chart
| Category | weight loss WAT |
|---|---|
